# “*Parenting my parents*”: Perspectives of adult children on assuming and remaining in the caregiver's role

**DOI:** 10.3389/fpubh.2023.1059006

**Published:** 2023-02-17

**Authors:** Jūratė Charenkova

**Affiliations:** Faculty of Philosophy, Institute of Sociology and Social Work, Vilnius University, Vilnius, Lithuania

**Keywords:** adult children, caregiving, wellbeing, choice, motivation, self-determination

## Abstract

**Background:**

Family caregivers are essential when responding to the long-term care needs of aging societies. The complex and multifaceted caregiver's role encompasses a unique set of challenges and strains, however, it can be a rewarding experience with many benefits and positive outcomes. Moreover, there is a link between the caregiver's wellbeing, quality of care, and the quality of life of the care recipient. Thus, the current study aimed to explore why adult children are assuming and remaining in the caregiver's role despite its challenges.

**Methods:**

Research data was collected through the use of qualitative semi-structured interviews from September 2021 to July 2022. In total 16 Lithuanian and Italian caregivers were recruited through convenience/snowball sampling. The study utilized the constructivist grounded theory for data analysis and self-determination theory for data interpretation.

**Results:**

Adult children's caregiving experiences revealed three themes related to the motivation to assume and continue with family care: (1) believing in the inherent value of family care; (2) making sense of the changing nature of caregiving; and (3) “*making the best of it*”. Key motivational drivers of these decisions were associated with the satisfaction of the three basic psychological needs – autonomy, competence, and relatedness. Results show that finding meaning and making sense of the caregiving role when responding to a parent's increased care needs may result in positive caregiving experiences and outcomes even at rather low levels of the care recipient's autonomy.

**Conclusion:**

Caregivers were able to experience family care as a meaningful and rewarding experience while acknowledging its challenges and limitations. Implications for family caregiving decisions and experiences, social policy, and future research are discussed in more depth in the paper.

## 1. Introduction

The rapid rate of population aging and an increase in the prevalence of long-term health conditions, such as dementia, has complex and far-reaching implications for all facets of society. Eventually, the health care and social services systems of many European countries will face major challenges in satisfying the long-term care needs of their aging populations. Family care is usually considered the most cost-effective long-term care option and is preferred by policymakers, older people themselves, and their family members (1, 2). Aging in place is prioritized before residential care and the goal of social policy often is to ensure that older people will stay in their homes for as long as possible (2). This trend is also relevant to many countries with strong family care traditions, like Lithuania and Italy.

Studies on the attitudes and expectations of society toward the care of older people in Lithuania reveal that almost 80 per cent of Lithuanians think family members should responsible for taking care of their aging parents (3–5). The attitude that children must take care of their parents in old age is also reinforced in Article 38 of the Constitution of the Republic of Lithuania: “the duty of children is to respect their parents, to take care of them in their old age, and to preserve their heritage” and in Article 3.205 of the Civil Code of the Republic of Lithuania: “adult children are obliged to provide for parents who have lost earning capacity and are in need of support”. These articles, however, were not yet enforced in legal practice. Additionally, there is hardly any support system or social services available for family caregivers at the state level (6, 7).

Similarly to Lithuania, in Italy, care for aging parents is considered to be a family matter with caregivers being entitled to some limited public support (e.g., the carer's allowance). This shapes the family care market, where assistance to dependent older people is generally provided by family members, especially adult children, and typically daughters (8, 9). Thus, most older people are cared for by families, even at rather low levels of autonomy (10). Consequently, family caregivers are essential when responding to the long-term care needs of aging societies, particularly in countries where family care dominates.

The complex and multifaceted role of the family caregiver encompasses a unique set of challenges and strains (11, 12). Caregivers are potentially at increased risk for adverse effects on their wellbeing in virtually every aspect of their lives, ranging from their health and quality of life to their social relationships and economic security. Thus, there is a large body of literature emphasizing various negative outcomes caregiving has on the caregiver's wellbeing and quality of life (13, 14). An increase in stressors is usually associated with worries about the care recipient's declining health, their controlling or manipulative behavior, and difficulties in managing care responsibilities with other work or family commitments (15–17). Additionally, restrictions on daily activities, free-time and social relationships can leave caregivers at greater risk of social exclusion in the future. A cumulative effect of these factors increases the risk of various physical and mental health issues and is associated with a unique type of stress – the caregiver's burden. These challenges are especially relevant if the person takes on the role of caregiver unprepared or feels pressured into it.

The connection between the perceived freedom of choice when taking on the role of a caregiver and various dimensions of emotional and psychological wellbeing, such as the level of caregiver's burden, or the quality of care provided, is studied extensively (18). Perceived lack of choice in becoming a caregiver is associated with higher levels of emotional stress, a negative impact on the caregiver's health, an increased risk of care breakdown, and a higher probability of nursing home relocation (18, 19). Additionally, adult children who feel that their autonomy in becoming caregivers was constrained might be unwilling to perform specific care-related tasks or acquire the knowledge and skills necessary to provide better quality care. This, in turn, is related to the higher risk of potentially harmful behavior toward the care recipient, such as abuse or neglect (12, 20). Thus caregivers' wellbeing and quality of life affect the quality of care they are providing, which in turn has an impact on the wellbeing and quality of life of the care recipient (19, 21, 22).

On the other hand, studies show that caregiving can also be a rewarding experience encompassing many benefits and positive outcomes. The most common caregiving rewards or benefits are potential for personal growth, enhanced self-efficacy, competence or mastery, self-esteem, satisfaction, and a sense of accomplishment (11, 12). Some of the positive outcomes include strengthening the relationships between caregivers and care recipients, which in turn has a positive effect on the quality of care (23, 24). Additionally, positive outcomes can mitigate some of the negative effects of caregiving, as several studies find that positive effects are associated with lower levels of burden, depression and psychological distress, and better overall mental health (23, 25). This means that caregivers can simultaneously experience both positive outcomes and negative impacts of caregiving. Thus, the actual outcomes for individual caregivers are both wide-ranging and highly individualized and depend on a variety of individual and contextual characteristics (19, 25).

The body of literature on the negative effects of caregiving, however, is far larger than that on positive effects, as researchers have sought to assess the public health implications of caregiving and identify vulnerable at-risk caregivers (25). According to Dombestein et al. (13), the present understanding of caregiving is still based on a stress-coping paradigm to reduce the burden on caregivers as the main goal of health services and social policy. Thus, the promotion of the positive aspects of caregiving, such as a sense of satisfaction, autonomy, and expertise among caregivers deserves greater attention.

The current body of literature on caregiving outcomes reveals that circumstances of assuming and motivation for remaining in the caregiver's role have an impact on how adult children will experience family care and what effect it will have on them. Thus, the current study aimed to explore why adult children are assuming and remaining in the caregiver's role despite its complex challenges. Additionally, knowledge about positive caregiving experiences can inform the policy and practice to better address caregivers and older people's needs, reduce the caregiver burden and foster the social services system that enables older people to stay in their homes for as long as possible. As caregivers' wellbeing and quality of life affect the quality of care they are providing, fostering their satisfaction with care can in turn increase the quality of life of care recipients.

By strengthening knowledge about the positive experiences of family care, this study can encourage older people and their family members to take a proactive approach when making long-term care decisions and facilitate their engagement when deciding on the care alternatives in the family. Additionally, social policymakers and health care professionals can develop more effective strategies and policies that improve the wellbeing of family caregivers and their aging parents, and reduce the burden of care, thus creating more favorable family care contexts. Such development of a sustainable long-term care system can foster opportunities for aging people to enjoy dignified and adequate support at the final stage of their lives, and can significantly reduce the burden of family care on adult children. Thus, the results of the study may be relevant to the health professionals working in the long-term care field, social policymakers, older adults, their family members, and other interested citizens.

### 1.1. Theoretical framework of the study

Ryan and Deci (26, 27) self-determination theory was employed to provide a theoretical lens for the research data. The theory posits that the subjective wellbeing, psychological growth, thriving, and successful functioning of a person depend on the conditions of their social context that can promote or prevent the healthy processes of psychological development. According to the theory developers, a person cannot achieve a sense of wellbeing if their social context does not provide opportunities to satisfy three basic and universal social needs: relatedness, competence, and autonomy.

The need for relatedness encompasses a feeling of mutual belonging and genuine connectedness with others – to experience giving support to and being supported by others. The need for competence relates to the ability to control the outcome and perception of performing tasks with confidence and effectiveness, and being capable of achieving desired outcomes – to experience mastery. And lastly, the need for autonomy relates to the sense of controlling one's life and being able to influence decisions – to be the causal agent of own life. In the context of self-determination theory, autonomy, however, does not mean independence from others, rather it refers to the ability to act in harmony with one's integrated self and implies the free will to act in accordance with one's values and interests. Thus, autonomy is about fostering a sense of choice and a feeling of having ownership of one's actions in contrast to a sense that choices and decisions are dictated by external factors. The sense of wellbeing is fostered in a social context that provides prerequisites to satisfy all three psychological needs. Thus, if the need for relatedness and autonomy is satisfied, but the need for competence is not, the person's ability to thrive in that particular social context will be compromised.

Satisfaction of the three needs also leads to the intrinsic motivation to pursue life goals, which in the case of family care, can translate into a positive attitude toward the caregiving role and an increase in adult children's wellbeing and quality of life. On the other hand, a restrictive social context disrupts the possibilities for self-expression, which can lead not only to a lack of initiative and responsibility toward the care recipient but also provokes stress that may cause both health and psychological issues to caregivers. In the context of caregiving, both countries participating in the research – Lithuania and Italy – have very strong and deeply rooted family care traditions. Studies [i.e., (11, 20, 21)] reveal that the sense of duty to care for one's parents relates to social-cultural norms and traditions. Consequently, trying to fulfill the socially conditioned duties of a child can limit the perception of choice when entering the caregiving role. Perceived lack of choice thwarts the satisfaction of the need for autonomy, which in turn, has a negative effect on the caregiver's wellbeing. Thus, self-determination theory may yield a better understanding of the effects caregiving has on adult children's wellbeing as well as the perception of their autonomy, competence, and relatedness in the context of family care.

## 2. Materials and methods

### 2.1. Research method and sampling

The study aimed to explore adult children's experiences of caring for their aging parents with a focus on their motives to assume and remain in the caregiving role despite its challenges. Family caregivers from two countries with a strong family care tradition – Lithuania and Italy – participated in the study. Lithuania's social services system is only 30 years old, with predominant family care and a lack of a governmental-level support system for family caregivers (5). On the other hand, in Italy, a social support system for family caregivers is more developed and most older people – even at rather low levels of autonomy – are receiving care from their family members (8–10).

As qualitative research embraces participant voices that accurately represent them and their experiences in an authentic form, this research design was deemed the most appropriate for the aims of the study. Additionally, a qualitative research format gives an opportunity to meet people in their natural environment, and to hear and see their social world through their voices and lenses (28). An in-depth, semi-structured interview method was used for the data collection because it enhanced the potential to explore authentic family caregiving experiences while creating a situation for caregivers to reflect on the effect taking care of the aging parents had on their lives.

Study participants were recruited through convenience/snowball sampling. In Lithuania, potential participants were first identified through the personal social network of the author (i.e., in the circle of friends, colleagues, or relatives) and by approaching associations uniting family caregivers in Lithuania (such as *Dementia Lithuania, Lithuanian Alzheimer's Disease Association* and similar). Additionally, each participant was asked to identify other caregivers who would be willing to take part in the study. Study participants were recruited from different Lithuanian cities (i.e., Kaunas, Vilnius, Šiauliai) and their districts.

In Italy, potential study participants were identified with the help of researchers from the Relational Social Work Research Center of the Catholic University of the Sacred Heart. Researchers identified and recruited potential study participants who met the inclusion criteria, briefly introduced them to the research idea, and asked, if they would like to take part in it. Those who agreed were then provided with detailed research information and aspects of informed consent. Italian study participants were recruited from three different Italian Regions (Trentino-Alto Adige, Lombardia, and Emilia-Romagna).

Several sample criteria were used to capture the diversity and nuance of the caregiving experience, such as different health statuses of the care recipient (caregivers of relatively independent parents and those whose parents have more profound disabilities), different duration of care provision, and the use of home help services (see Table 1 for sample characteristics). In total 16 Lithuanian and Italian family caregivers participated in the research.

**Table 1:** Sample characteristics.

**Code**	**Adult child's gender/age**	**Length of care**	**Relation to the care recipient**	**Age of care recipient**
LT1	Female/67	15	Mother	91
LT2	Male/50	5	Father	75
LT3	Female/53	10	Mother	74
LT4	Male/44	2	Both parents	74 (father) 69 (mother)
LT5	Female/46	10	Both parents	76 (father) 81 (mother)
LT6	Female/68	12	Mother	88
LT7	Female/64	8	Mother	83
LT8	Female/57	7	Mother	78
IT1	Female/53	5	Mother	75
IT2	Female/50	10	Mother	83
IT3	Male/59	1,5	Mother	82
IT4	Female/49	7	Mother	75
IT5	Female/48	13	Father	84
IT6	Female/72	5	Mother	93
IT7	Female/67	1	Mother	89
IT8	Female/62	5	Mother	86

### 2.2. Data collection and analysis

Research data was collected through the use of individual qualitative semi-structured interviews with 8 Lithuanian and 8 Italian caregivers. At the beginning of the interview, study participants were asked to narrate their caregiving history from the very beginning. After the initial story, more questions followed detailing the circumstances of assuming the caregiver role, changes in lifestyle after becoming a caregiver (i.e., daily routine, positive outcomes and restrictions of this role), and care-related future plans. Depending on each interview, some questions may have been changed, refined or additional questions asked to provide more insight into the adult child's caregiving experience. The first pilot interviews with two caregivers were held in September 2021. These interviews served as a test and helped to further develop and refine the interview guide for the research.

The study utilized Charmaz's (29) version of grounded theory for the analysis of the data. With the consent of the study participants, all interviews were recorded using a voice recording app on the smartphone and later transcribed verbatim. As Charmaz (29) does not offer specific transcription rules, thus in the transcripts pauses of silence, changes in intonation, emotions or specific expressions of the study participants were marked according to the common transcription guidelines. When transcribing the conversations, an attempt was made to write in the normative language with the usual characters of the written language, however, the authentic expressions or narration of the research participants in another language were not omitted or corrected. Although in the analysis of the interviews detailed and thorough transcripts were used, when citing in this paper repetitions or interruptions of thought were removed without changing the essence of the narration to make the text more understandable for a reader.

Transcriptions were coded using MaxQDA 10 software designed for qualitative research. The shortest interview lasted 35 min, the longest was 1 h 23 min. The record of all interviews was about 13 h. Interpretation of the research results was based on the concepts of self-determination theory (26, 27).

### 2.3. Research ethics

Privacy and confidentiality were critical issues in gaining the trust and confidence of the study participants. Several interviews were conducted *via* phone, and others – were face-to-face. The study adhered to the key ethical principles of qualitative research. All interview sessions were conducted with no third-party involvement in a relaxed and interactive manner resembling a friendly conversation. Study participants shared their experiences in an open manner and at the end of the interview, many were surprised that the duration of the conversation was longer than they initially anticipated.

Interviews were individually recorded with participant consent and participants were ensured of the confidentiality of the information. During the data analysis stage, all information that could help identify study participants (i.e., names of people and places, or other sensitive information) was removed. This qualitative research employed in-depth interviews as part of broader postdoctoral research funded by the European Social Fund under an agreement with the Research Council of Lithuania. The ethical approval for the research was obtained from the Ethics Commission of the Department of Social Work and Social Welfare of Vilnius University in September 2021.

## 3. Results

Lithuanian and Italian adult children's caregiving experiences revealed three themes related to the motivation to assume the caregiver's role and continue to provide care to their aging parents: (1) believing in the inherent value of family care; (2) making sense of the changing nature of caregiving; and (3) “*making the best of it*”. Each theme will be discussed below in more detail.

### 3.1. Believing in the inherent value of family care

Although care expectations were never explicitly discussed between family members, taking care of parents in old age was an obvious choice for all caregivers. The decision to provide care at home was often influenced by the close relationship between adult children and parents. In many cases the decision to assume the caregiving role was motivated by a caregiver's desire to repay parents for all the love and support they received in childhood: “*parents provide for you, they give you an education, they give you everything they have best: their health, their time, their money, of course, you have to take care of them, it couldn't be otherwise*” [LT1]. In the context of self-determination theory, the feeling of genuine connectedness with aging parents and the experience of providing support when they are in the need satisfied the caregiver's need for relatedness, thus promoting their sense of wellbeing.

Family care was perceived as a way to fulfill a duty of a child: “*for me, as a daughter, it was a must to take care of my mum*” [IT4]. Caregiving as a duty of women and daughters was particularly emphasized in the narratives of Italian female caregivers: “*As a daughter and as a woman, I feel closer to my dad compared to my brother, I think that women are simply able to sense things better than men, their sense of empathy is stronger, and they have more compassion*” [IT8]. Thus, in addition to the sense of emotional closeness to one's parents, the caregiver's sense of wellbeing was further supported by the belief that one is able to perform certain tasks better either because of the skills or character traits. Maintaining this belief supports the sense of confidence of being able to provide good quality care – to experience mastery, which contributes to the satisfaction of the need for competence.

It is possible that due to the strong family care tradition in the Lithuanian and Italian cultures, caregivers saw taking up the responsibility to care for their aging parents as something that was both self-evident and expected of them and they did not reflect much on this decision. To many caregivers “[taking care of parents at home] *was a natural solution*” and “[they] *didn't really think much of it*” [LT3]. They mentioned that the alternative of not being involved in the care of aging parents and instead turning to other options (i.e., residential care) never crossed their minds. Caring for parents at home was seen as an act that has a high value and showcases one's high morals. Especially in Lithuanian interviews family care was held in very high regard and considered morally superior compared to residential care. Children who refuse to care for their parents were usually frowned upon while their parents pitied.

One caregiver shared in detail a story of a neighbor whom her daughter “*gave away*” to the nursing home. In the caregiver's narrative, such a decision made the daughter not only a ‘bad daughter', but also a ‘bad person' who failed to appreciate all the sacrifices her mother made while raising her: “*If you love* [your parents] *you will take care of them* [at home]*, it's a living person, not a thing* < …> *they took care of you for your entire life, fed you, dressed you, loved you, you can't just leave them like that!*” [LT1]. Another research participant shared that he would have felt very guilty if his parents would live in the nursing home. However, in his narrative family members who don't provide care for their parents were not villainized for such a decision:

*I don't judge those, who give away* [their parents], *I think my situation was very favorable, as both my parents were still in their right minds, but when a person is bedridden, and can't feed themselves, or even can't use the bathroom themselves* < …> *you never know, what story that family had, you can't judge them* [LT4].

Negative attitude toward residential care was further reinforced by the belief that the home environment has a positive effect on the wellbeing of aging parents: “*mom is holding on only because she is at her home, she knows every corner here, every brick while* [in the nursing home] *she will be surrounded by a bunch of strangers*” [LT3]. Thus, the decision to provide care at home always reflected the caregiver's innate values and beliefs about the proper care of aging parents. These beliefs were also in line with the societal attitudes toward the long-term care of older people meaning that caregivers have internalized the cultural norms and did not feel pressured into assuming the caregiving role. Consequently, the belief that one simply does what is perceived as morally right and lives up to the social and cultural expectations related to family care satisfied the caregiver's need for autonomy. Thus, the belief in the inherent value of family care and its positive effect on aging parents often encouraged adult children to continue with home care even if their parents' autonomy decreased significantly.

### 3.2. Making sense of the changing nature of caregiving

Caregiving was often experienced as a journey that did not have a clearly defined beginning. Many caregivers reflected on becoming involved in their parents' care gradually and somewhat organically: “*we all saw* [parents] *needed more help – it's just a part of aging, so of course, it's only natural, you are not of the same strength nor in the same health as in your youth*” [LT5]. The help initially was mostly instrumental, such as buying groceries, paying taxes, or cleaning around the house. However, due to the aging-related health decline, parents eventually become more dependent on their children. The caregiving duties expanded significantly: “*Mom needs help with everything now, I help her to get up from the bed, to rise from the chair, to get dressed, to get her hair done* < …> *I bathe her, I help her with all the hygiene stuff* ” [IT6].

Witnessing parents' gradual physical and cognitive health decline altered caregivers' views on old age. Many reflected that while older people in modern societies are living longer than ever, their aging experiences can be very different. Some older adults may be playing golf, and be relatively healthy and cheerful, while others can be bedridden and suffer from various health issues (i.e., dementia or Alzheimer's disease). Following this realization, many family members reconsidered the meaning of caregiving. As parents' care needs increase, caregiving duties may transition from drinking tea with a parent and reminiscing to taking care of a bedridden and confused parent who does not recognize the caregiver as their child. A daughter, who took care of both of her parents reflected: “*One day I just had this epiphany – you need to bathe them, to change their diapers, to feed them, it's like taking care of a child, but it's your parents* < …> *from a daughter I become a mother to my parents*” [LT5].

Although the notion of ‘parenting [one's] parents' was initially upsetting and frightening to many adult children, caregiving was nevertheless considered a meaningful task. One caregiver reflected: “[parents'] *life, although it may appear fruitless and devoid of meaning, is actually very meaningful, as they are heading toward the end of their days* < …> *you really can't do anything but to accompany them there*” [IT7]. Knowing that they are supporting parents in the last and most vulnerable stage of life strengthened caregivers' sense of self-esteem and belief that they are successfully fulfilling their duties as children, thus supporting their need for autonomy. Additionally, ensuring their parents' dignified accompanying toward the end of their lives often motivated caregivers to continue with family care as many were convinced that their parents will not receive the same level of care, love, and support in the nursing homes. This notion was particularly emphasized in the narratives of Lithuanian caregivers. One daughter elaborated:

*The nursing homes are always awfully crowded, I think we can safely assume the level and quality of their services* < …> *the staff is tired and overworked, they have their own things going on, and they will for sure not have time for* [mother] *although she is very willing to communicate* [LT1].

Additionally, caregivers were worried that nursing home staff will fail to see their parents as unique people or will not understand them: “*I take care of my mum for almost 10 years now, I know her look, her smile, her touch, and to* [nursing home staff] *she will be just another old lady*” [LT3]. Thus, there was a certain sense of pride in being able to provide for one's parents and to understand them better than anyone else: “*No one knows my mum better than me and no one will take her of care better than I will*” [LT6]. Fostering a close relationship with parents and having an intimate knowledge of the peculiarities of their behavior strengthened the caregiver's belief that they will be able to successfully cope with difficulties even as parents' care needs increased significantly. These notions feed in the satisfaction of the needs for relatedness and competence.

### 3.3. “Making the best of it”

Taking care of one's aging parents was universally considered a duty that requires a lot of time, effort and devotion. With parents' care needs increasing, caregiving becomes even more complex and demanding. Some adult children reflected on how over time caregiver's role grew increasingly immersive:

*You are essentially bound* < …> *it doesn't matter, what's the weather today or how's your mood, you still have to clean around the house or cook food, you can't say “Oh, I just don't feel like it today*”*, there are things that you must do and you do it* [LT1].

Due to the ‘bounding' nature of the caregiving role, many noticed that eventually, the amount of time to dedicate to oneself decreases significantly. Thus, caregivers reflected on the importance of “*maintaining the balance*” [IT8] between providing care and taking care of oneself. Fostering a positive state of mind, having an awareness of own limitations, and taking breaks were frequently mentioned as important measures to prevent burnout and overcome a sense of being consumed by the caregiver role. In the context of self-determination theory, employing these coping techniques relates to the satisfaction of the need for autonomy. Choosing when and what caregiving tasks to perform, when to provide care for one's parents, and when to take care of oneself strengthens the feeling of satisfaction with family care and a sense of control of one's life.

Interestingly, Italian caregivers often mentioned group support as means to “*replenish* [their] *inner resources*” [IT4], such as meeting with friends and attending religious circles or family caregivers' support groups. Lithuanian caregivers' narratives, on the other hand, revealed a sense of loneliness when providing care. Although other family members, friends, or neighbors in some cases were seen as potential helpers, caregiving was mostly perceived as a solitary task: “*I mean, I know they* [brother and his wife] *could help if I would ask, but I am mostly alone in this*” [LT6]. The sense of being alone in the context of self-determination theory diminishes the satisfaction of the need for relatedness as caregivers don't feel supported by others. Although Lithuanian caregivers mentioned feeling appreciated by family members, they felt that as soon as they became primary caregivers, others stepped down: “*you constantly need to ask for help, it's like people don't even think about maybe asking if you need something*” [IT7].

Additionally, caregivers mentioned the importance of “*living one day at a time*” [LT4] and solving issues (relational, behavioral, and practical) as they arise instead of planning ahead. Focus on the present as a possible coping strategy was particularly noticeable in the caregivers of parents in the later stages of dementia. As one daughter reflected: “*It becomes increasingly difficult to plan something if you think about it – anything could happen, but not everything will happen, so I try to live in the here and now, it's less confusing this way*” [laughs] [IT5].

Caring for one's aging parents may have its own unique challenges, however, to many adult children, it was a continuous and authentic learning experience with many opportunities for personal growth. Caregivers reflected gaining a variety of everyday skills and learning new things that were simply not needed earlier in life, such as learning how to drive, change a light bulb, or do small home repairs. In addition to this, many obtained new competencies related to caregiving, particularly dementia care as due to disease, verbal communication was often limited and parents not always were able to recognize their children. Caregivers learned to appreciate rare moments of lucidity and enjoyed being able to get a sense of relationship reciprocity while acknowledging the diminishing occurrence of these moments. In some cases, the caregiver's role strengthened the relationship with the parents, even though the relationship was not that close earlier:

*I felt closer to my mum than my dad my entire life as he always seemed somewhat emotionally detached, but caregiving bonded us in completely different and new ways, and now we are closer than ever* < …> *through* [caregiving] *I think I am now finally able to see and appreciate the unique ways he always showed his love to me* [IT5].

The confidence that successfully solving a current problem will make one able to efficiently solve future problems boosted caregivers' self-esteem and helped them feel more confident about the future, thus satisfying the need for competence. Additionally, learning new ways to meaningfully connect with parents strengthened the parent-child bond and helped to satisfy the need for relatedness.

## 4. Discussion

The study aimed to explore adult children's experiences of caring for their aging parents with a focus on their motives to assume and remain in the caregiving role despite its challenges. Key motivational drivers of these decisions were often associated with the satisfaction of the three basic psychological needs – autonomy, competence, and relatedness. These needs are essential for fostering the caregiver's motivation to provide care for their aging parents and maintaining their sense of wellbeing. Consequently, while acknowledging the emotional and physical toll of taking care of one's aging parents, adult children expressed a strong desire to continue with family care even at rather low levels of care recipient autonomy.

Satisfaction of the need for relatedness encompassed the sense of being acknowledged, appreciated, and supported by the closest family members, friends, and the aging parents themselves while performing caregiving tasks. A feeling that one is appreciated for taking care of parents and having their struggles acknowledged made caregivers feel more enthusiastic and motivated to continue with family care. Obtaining new skills or knowledge necessary to perform caregiving tasks more effectively fostered a sense that one is able to successfully cope with care-related challenges. This allowed adult children to experience mastery, which in turn led to the satisfaction of their need for competence. Particularly in dementia care, feeling that one is able to ensure the aging parent's wellbeing was an especially important motivational driver to continue with family care. Additionally, it appears that many caregivers are motivated to obtain new skills if they believe this will help to better respond to the parent's needs. In the case of Italian adult children, many of whom belonged to various emotional support groups for dementia caregivers, the need for competence could be satisfied together with the need for relatedness. As members of support groups, these caregivers were able to foster a sense of belonging to the group and at the same time obtain important information or useful advice from other dementia caregivers.

The need for autonomy in the context of this study was related to volition and a sense of freedom while choosing to take on the caregiver's role or performing certain caregiving tasks. Exerting control over the extent, duration, and contents of caregiving duties contributed greatly to the sense of wellbeing of the caregivers and their satisfaction with the care. Additionally, the need for autonomy was strengthened by a belief in the inherent value of family care and in the positive outcomes the home environment has on the wellbeing of an older parent. All research participants claimed that assuming the caregiving role was their own choice and that they never considered the option of not taking care of their parents. Maintaining the balance between taking care of parents and taking care of oneself was an important strategy to prevent burnout.

Although most people are willing to take on the role of caring for a loved one, the options for taking on the role of caregiver can vary, with some adult children taking on the role actively, and others accepting it passively or having the role assigned to them (21, 30). Thus, the adult children's decision to take care of their aging parents can be a free choice or a choice that is constrained by various circumstances, such as a sense of duty, prevailing societal expectations about family care, the lack of financial resources or insufficient social support (11, 20).

In this study, both Italian and Lithuanian caregivers often mentioned the importance of social or cultural norms about family care in their decision on assuming a caregiver's role. Nevertheless, they did not think of themselves as being pressured into the role and often felt empowered by the sense of doing what was perceived as morally and culturally right in society. Similarly to Dombestein et al. (31) study, it appears that adult children have internalized the notion of the inherent value of family care and assumed a caregiving role without thinking too much about their autonomy in the decision-making process. Becoming a caregiver was always seen as a voluntary act, that might be motivated by a desire to repay parental love in childhood, a sense of duty, or a belief that one can take care of a parent the best. It was never seen as a decision dictated by external factors. Thus, knowing that one is keeping their parents in the comfort of their homes and fulfilling societal norms of filial duties was essential for the decision to continue with family care and for the satisfaction of the need for autonomy.

All caregivers considered caring for aging parents a child's duty, however, in some cases, the desire to do everything on one's own can create a potential for burnout. Some believed that relying on others for support might be perceived by others as a sign of them failing in their caregiving duties. Thus, some caregivers tend to avoid seeking help or considering other care alternatives (for example, home-help services or nursing homes). The attitude of overcoming all difficulties on one's own, no matter what obstacles arise, can, in the long run, contribute to the stress experienced by caregivers and lead to feelings of burnout, which in turn can negatively affect the quality of care and therefore the quality of life of parents (19, 22). This tendency eventually can create unique challenges when organizing the health and social services systems in a way that fully meets the needs of a growing older population and their caregivers, especially when the independence of an older person decreases.

Assuming the caregiving role and continuing to provide care to their aging parents when responding to a parent's increased care need may result in positive caregiving experiences and outcomes even at rather low levels of the care recipient's autonomy. Thus, it is essential to support caregivers' motivation and their wellbeing, which in turn, affects the quality of life of a care recipient. The results of this study indicate that additional quantitative and qualitative studies need to be undertaken with larger sample sizes to fully capture the diversity and nuance of the caregiving experience and outcomes. Future research could examine how societal attitudes and other macro-level factors affect adult children's caregiving experiences and motivations. Studies focusing on social and cultural norms related to family care and gender roles could contribute to the care research on the perspectives of men caregivers. Additionally, comparing the perspectives of older people and their adult children would allow a deeper understanding of factors affecting their wellbeing and life satisfaction.

This study has few limitations. Firstly, as in all qualitative studies, the relatively small and local sample represents an obvious limitation. Caregivers from two countries participated in this study and although data is rich and reveals nuanced caregiving experiences, a larger nationwide sample size would have made this research more focused and better suited for generalizability. Secondly, although the research participants were from different socio-economic backgrounds and had various caregiving experiences, the sample is not representative of the entire Lithuanian or Italian population of family caregivers. Moreover, the male perspective is not comprehensively addressed in this study. This underrepresentation, on one hand, reflects that family care is mostly provided by women, on the other hand, societal norms regarding the care of aging parents also usually emphasize the social role of women. Thus, it's worth pointing out that son caregivers' experiences are likely different from daughters. Thirdly, the unique socio-cultural context, social services organization system, and national support systems for the family caregivers need to be considered when analyzing the care decision-making processes and caregiving experiences. Family care tradition is very strong both in Lithuania and Italy, thus, caregiving experiences in these countries can differ from the experiences in countries with weaker family care traditions, more developed home help services, or established support networks for family caregivers. Additional quantitative and qualitative studies need to be undertaken with larger sample sizes to capture the diversity of the caregiving experience, its influencing factors and positive outcomes.

## Data availability statement

The datasets presented in this article are not readily available because interviews consist of sensitive personal data (such as religious beliefs, family relationships history, etc.) and only anonymized data is used for the purposes of this study. Requests to access the datasets should be directed to JC, jurate.charenkova@fsf.vu.lt.

## Ethics statement

The studies involving human participants were reviewed and the ethical approval for the research was obtained from the Ethics Commission of the Department of Social Work and Social Welfare of Vilnius University in September 2021. The patients/participants provided their written informed consent to participate in this study.

## Author contributions

The author confirms sole responsibility for the study conception and design, data collection, analysis, interpretation of results, and manuscript preparation.
